# A dynamic evaluation of macao environmental policies based on text mining of the PMC knowledge framework

**DOI:** 10.1371/journal.pone.0336786

**Published:** 2025-11-18

**Authors:** Rongjiang Cai, Tao Zhang, Xi Wang, Shufang Zhao, Qiaoran Jia

**Affiliations:** 1 School of Economics and Management, Ningbo University of Technology, Ningbo, China; 2 Faculty of Humanities and Social Sciences, Macao Polytechnic University, Macao, China; Macau University of Science and Technology, MACAO

## Abstract

This study analyzes policy statements issued by the Macao Special Administrative Region government since the handover, employing text mining methods to explore the trajectory of Macao’s environmental policy development. Results indicate that the Macao government has continuously increased investment in ecosystem resources and implemented diversified strategies to establish a comprehensive environmental management system. Furthermore, the government has integrated environmental policies with broader national development strategies, emphasized cultivating environmentally responsible talent, and focused on key areas such as climate change adaptation, pollution prevention and control, ecosystem protection, and sustainable urban development. This study applies the PMC index model for quantitative analysis, using 9 major variables and 50 minor variables to evaluate important environmental policies implemented between 2010 and 2024. Through the combination of text mining and quantitative analysis with the PMC index, detailed evaluations of these policies are provided. With all policies achieving perfect scores in policy evaluation and disclosure but showing weaknesses in timelinessand regional scope.The research utilizes three-dimensional PMC surface diagrams to visualize policy strengths. Additionally, employing multi-dimensional input-output table analysis methods, the study assesses the overall performance of each policy, identifies areas requiring improvement, and offers targeted recommendations. Macao’s environmental policies demonstrate strong transparency and evidence-based design but require enhanced long-term strategic planning and cross-border cooperation mechanisms within the Greater Bay Area framework..

## 1 Introduction

As one of the four central cities in the Guangdong-Hong Kong-Macao Greater Bay Area, Macao plays an extremely important role in driving prosperity and development in Guangdong Province, Hong Kong, and other regions. Its development and stability are closely related to China’s reform, opening-up, and modernization process [1]. As the foundation of social development, the environment has had a significant impact on Macao’s social progress and the long-term implementation of the “One Country, Two Systems” policy. Against the backdrop of globalization and regional integration, adjusting and strengthening Macao’s environmental policies is crucial for optimizing its social structure. These adjustments are extremely important for promoting regional integration and enhancing competitiveness [2,3]. Current research on Macao’s environmental policies primarily focuses on key areas such as climate change mitigation, pollution prevention and control, ecosystem protection, preservation and restoration of urban ecological spaces, solid waste management, sustainable urban development, strengthening environmental legal systems, and regional cooperation and exchange [4,5]. However, these studies are mostly limited to descriptive analysis, lacking comprehensive assessment of policy implementation and their social impacts [6–8].

Although existing literature explains Macao’s environmental policies from multiple perspectives, it does not adequately address the specific impacts and effectiveness following policy implementation [9]. Systematic research on the impact of environmental policies on socioeconomic development and the role of environmental reform in promoting regional integration remains limited. Additionally, existing research relies heavily on qualitative analysis, with limited scope for quantitative assessment [10]. This study aims to fill these gaps by conducting a comprehensive analysis of the historical evolution of Macao’s environmental policies and their social impacts. Through systematically investigating post-handover policy documents and employing text mining techniques and quantitative analytical tools such as the PMC index, this research evaluates the effectiveness of these policies [11,12].

By constructing multidimensional data models and developing an assessment framework that integrates qualitative and quantitative analyses, this study provides new perspectives for understanding and evaluating Macao’s environmental policies. Methodologically, this research employs the PMC index and text mining techniques, introducing scientific and systematic policy analysis methods [13,14].

This study aims to conduct a comprehensive, quantitative evaluation of Macao’s environmental policies (2010–2024) by integrating text mining and the PMC index model, providing empirical evidence for policy optimization.“

RQ1: What are the core themes and priorities in Macao’s environmental policies?
RQ2: How consistent and comprehensive are these policies in design and implementation?
RQ3: What are the specific strengths and weaknesses requiring policy intervention?

The objectives of this study include: (1) quantitatively assessing the effectiveness of Macao’s environmental policies, (2) identifying policy strengths and areas for improvement, and (3) serving as a reference model for environmental policy formulation in other similar contexts. This will address the lack of empirical and systematic policy evaluation in existing literature.

## 2 Literature review

### 2.1. Evolution of environmental policy evaluation

Environmental policy assessment has long been an important means for understanding policy effectiveness and impact. Early research primarily relied on qualitative descriptions and case studies, which had limitations in capturing the complex interactions of policies. Since the 1990s, assessment methods have become more sophisticated, introducing quantitative approaches. For example, Dijk et al. (1996) developed an integrated framework using micro-simulation models (NFM) combined with agricultural census data to evaluate environmental policies. This highlighted the importance of considering the complex interactions between economic activities and the environment in policy assessment [15]. Similarly, Frenken et al. (2004) examined the development of environmentally friendly vehicle technology using patent data, underscoring the importance of technological and organizational diversity in advancing environmental technological innovation [16]. Such models are powerful in predicting potential outcomes of policy interventions but typically require large-scale datasets and specialized expertise.

### 2.2. The rise of computational methods in policy analysis

In recent years, with the explosive growth of digitized textual data, computational methods, especially text mining, have gained significant attention in the field of policy analysis. Text mining is the process of exploring essential patterns and associations from large volumes of unstructured textual information and extracting valuable insights. By analyzing textual data such as policy documents, meeting records, and media reports, researchers can systematically identify themes, sentiments, and trends that human readers might easily overlook. For instance,.Rodriguez et al. (2008) used text mining to quantitatively evaluate China’s new energy vehicle policies and extracted key topics and trends from policy documents. This approach provides an objective and replicable pathway for tracking temporal changes in policy discourse and prioritiesutilize [17]. The robust mathematical planning technique proposed by Doole and Pannell (2011) provides a novel solution to address the issue of inadequate information in environmental policy assessment [18]. Recent advances in natural language processing have enabled more sophisticated thematic extraction from policy documents. Danesh et al. (2021) [19] demonstrated the effectiveness of Latent Dirichlet Allocation (LDA) for thematic analysis in large document corpora, providing a retrospective and prospective framework for understanding topic evolution over time. Similarly, Danesh and Dastani (2023) [20] illustrated how Python-based NLP techniques can systematically extract patterns from historical document collections, offering methodological insights applicable to policy text analysis.

Additionally, Wegner and Pascual (2011) criticize the constraints of cost-benefit analysis tools and advocate adopting a pluralist framework to assess policy impacts comprehensively [21]. Regarding the quantification and systematization of policy assessment, Bel and Holst (2018) introduce a quantitative tool for evaluating transport policies [22]. This tool analyzes the effects of bus rapid transit (BRT) systems using difference-in-differences techniques. Subsequently, Kuminoff (2018) introduces a conceptual model of consumer categorization in the housing, labour, and healthcare markets and discusses the application of benefit transfers, offering a new theoretical framework for examining consumer behaviour [23]. Furthermore, Costa et al. (2018) utilize an epidemiological quasi-experimental statistical approach to evaluate the influence of land-use policies on deforestation rates, providing a new statistical methodology for assessing land-use policies [24]. Zeiger et al. (2019) delineate a framework for evaluating sustainable development endeavours, measuring environmental planning systems and environmental outcomes, offering a comprehensive framework [25].

### 2.3. Index models for policy assessment: the PMC framework

While text mining excels at exploratory analysis of policy content, assessing the quality and consistency of policy design itself requires a more structured framework. Here, the Policy Modeling Consistency (PMC) index model emerges as an effective tool. Developed by Schoenefeld and Jordan (2019), the PMC index is a quantitative policy assessment method used to evaluate the strengths and weaknesses of policy documents [26]. The model decomposes policies into multidimensional variables (such as policy types, objectives, constraints, incentives) and assesses each variable with binary values (0 or 1) based on its presence or absence. As it does not require subjective weighting, this method reduces subjectivity in the assessment process and facilitates comparisons between different policies.

Yimsuk and Thammaboosadee (2024) applied the PMC index model to quantitatively assess China’s farmland protection policies, revealing their effectiveness and internal consistency. This approach objectively demonstrates the comprehensiveness and logical consistency of policy design [27]. Regarding empirical research and model development for policy assessment, Chen Die et al. (2019) developed an economic policy assessment model to analyze network car policies, providing a new framework for policy assessment within the emerging economic paradigm [28]. Similarly, Hynes and O’Donoghue (2020) employed a novel value function transfer method to evaluate eco-efficiency, introducing a new quantitative tool [29]. Moreover, Kuang et al. (2020) assessed the effectiveness of arable land protection policies utilizing the PMC-Index model, introducing an innovative quantitative approach for evaluating the efficacy of these policies [30]. Bouma (2021) conducted an extensive review of the utilization and value of experimental methods in environmental policy assessment, highlighting the potential of employing mixed methods and providing theoretical underpinnings for advancing innovative policy assessment methods [31].

Similarly, Chen et al. (2021) utilized the PMC index model to screen policy texts and develop a model for assessing the support of policy measures, offering an innovative model for quantitative analysis of policy texts [32]. Tan and Mao (2021) examined the influence of the central environmental protection inspection policy on air quality, providing empirical analyses for evaluating environmental regulatory policies [33]. Mentzakis and Sadeh (2021) offer a new experimental economics perspective for evaluating environmental economic policies, examining the effects of incentives on risk aversion and discounting tasks for monetary and environmental goods [34].

Regarding the systematization and quantification of policy evaluation, Lu et al. (2021) introduce a new systematic approach to evaluating public health emergencies by analyzing the resumption policy during an epidemic in China using a policy evaluation model [35]. Similarly, Yang et al. (2021) utilized text mining to quantitatively evaluate China’s new energy vehicle policy, developing an innovative text analysis tool for assessing new energy vehicle policies [36]. Dai et al. (2021) evaluated the green development policy of the Yangtze River Economic Belt, proposing theoretical recommendations for enhancing regional green development policies and offering theoretical support for optimizing such policies [37]. Meanwhile, Brouwers et al. (2022) investigated the understanding of impacts among participants during the assessment of nature policies in the Netherlands, providing a fresh perspective on participant analysis in the policy assessment process [38]. Robson-Williams et al. (2022) utilized a causal framework to evaluate water resources management, proposing an innovative framework for assessing international water resources management policies [39]. Meanwhile, Peng et al. (2022) assessed the sustainability of long-term care insurance in China, offering a fresh perspective for the long-term assessment of social security policies [40]. Lastly, regarding the integrative and strategic aspects of policy assessment, Ferraro and Miranda (2014) utilize statistical techniques to replicate the results of water use experiments, offering a new statistical approach to assessing water management policies [41].

### 2.4. Integration and identification of research gaps

Based on the existing literature review, environmental policy assessment has evolved from qualitative methods to diverse quantitative approaches including micro-simulation, text mining, and the PMC index. However, the integrated application of these methods, particularly text mining and the PMC index, remains a relatively new field. Geng et al. (2015) employed this integrated approach to evaluate the scientific rigor of China’s wind power industry policies and proposed a new analytical framework. This study further develops existing research by applying this integrated approach to the unique context of Macao’s environmental policies [42]. Macao’s policies are influenced by special factors such as its status as a Special Administrative Region, highly urbanized environment, and role in the Guangdong-Hong Kong-Macao Greater Bay Area. To date, research on Macao’s environmental policies has been primarily descriptive, without systematic and quantitative studies assessing their design quality and effectiveness. Therefore, this study aims to fill this research gap by empirically identifying the main themes and priorities of Macao’s environmental policies through text mining and utilizing these results to construct and validate variables for the PMC index model. This hybrid research methodology promises to provide a more comprehensive and nuanced understanding of the strengths and weaknesses of Macao’s environmental governance.Additionally, Xu et al. (2015) proposed an integrated environmental policy, including measures such as constructing biomass power plants based on a dynamic planning model, introducing a novel approach to the systematic design of environmental policies [43]. Kunstler and Vasileiadou (2016) explored the effects of using multiple assessment methods concurrently on daily assessment practices, offering practical guidance for method selection and application [44]. Meanwhile, Medeiros (2017) conducted a geographical analysis of the impact of EU cohesion policy, providing empirical insights into the geographical variability of regional policies [45]. Additionally, Yang et al. (2017) utilized a qualitative approach to evaluate the effectiveness of policy initiatives by exploring farmers’ perceptions, introducing a novel psychosocial dimension to policy assessment [46]. Pereda and Lucchesi (2022) proposed two frameworks from a global holistic perspective to evaluate mitigation policies in the maritime sector, offering a fresh outlook on assessing global climate change policies [47]. In a related context, Yang et al. (2022) introduced a novel quantitative tool for evaluating traditional medicine policies based on the PMC index model, enabling a comprehensive assessment of policies related to the development of traditional Chinese medicine [48]. Wang and Xing (2022) also made a significant contribution to policy assessment by integrating text mining techniques with PMC index models to evaluate the scientific rigour of policies derived from China’s wind power industry policies, introducing a new framework for assessing renewable energy policies [49]. Additionally, Liu and Zhao (2022) developed indexes for assessing the effectiveness of textile industrial policies based on document analysis, integrating theory with the PMC index model and introducing a novel quantitative method for evaluating traditional industrial policies [50].

The review of existing literature reveals that prior research efforts have often utilized various quantitative techniques such as microsimulation models, PMC indices, and mixed-method approaches to assess environmental policies, providing diverse perspectives on policy impact [51]. However, the application of these techniques to Macao’s environmental policies remains limited. This study builds upon the foundations laid by these works, employing the PMC index model for consistency evaluation and integrating text mining for qualitative analysis. Specifically, the quantitative approach adopted here draws from Kuang et al.’s (2020) [52] use of the PMC-Index for cultivated land policies, while the integration of text-mining follows the methodology introduced by Yang et al. (2021) [53] for evaluating China’s new energy policies. Together, these approaches are adapted to address the unique context of Macao’s environmental landscape, incorporating insights from prior studies that emphasize the value of mixed methods to achieve a comprehensive policy assessment.

### 2.5 research status of macao’s environmental policies

#### 2.5.1. Descriptive studies on Macao’s environmental governance.

Existing research on Macao’s environmental policies has primarily focused on descriptive analyses of specific environmental challenges. Jin et al. (2006) [54] conducted seminal work examining solid waste management practices in Macao. More recently, Lan et al. (2025) [55] developed predictive models for municipal solid waste generation, highlighting the impacts of household dynamics and tourism-related factors on waste management challenges. Song et al. (2016) [56] explored residents’ attitudes and willingness to pay for solid waste management improvements, finding that 95.7% of respondents were willing to participate in waste sorting if required by the government. In the air quality domain, Lei et al. (2019) [57] contributed significantly to understanding Macao’s air pollution patterns through statistical forecasting methods, noting that nitrogen dioxide (NO2), particulate matter (PM), and ozone (O3) frequently exceed acceptable thresholds. Regional studies have documented that the Guangdong-Hong Kong-Macao Greater Bay Area underwent dramatic urban climate changes over the past four decades, with urbanization significantly altering material and energy exchange between surface and atmosphere. The Macao SAR government’s Environmental Protection Planning (2010–2020) established a comprehensive framework with three principal themes: “Optimizing the Environment Suitable for Living and Tourism,” “Promoting a Conservation and Recycling-oriented Society,” and “Integrating into the Green and Quality Region” However, these policy documents primarily outline objectives and strategies without systematic evaluation mechanisms.

#### 2.5.2. Regional cooperation research in the Greater Bay Area.

Macao’s environmental governance cannot be understood in isolation from its regional context within the Guangdong-Hong Kong-Macao Greater Bay Area (GBA). Du and Loh (2020) [58] analyzed climate change policies in Hong Kong and Macao, noting that while both Special Administrative Regions enjoy high policy autonomy with some aspects more advanced than mainland China, this autonomy has also isolated them from China’s larger-scale environmental management practices. Yu et al. (2024) [59] demonstrated that guided by government policies, interactions among economic, environmental, and resource elements in the Greater Bay Area continue to strengthen, highlighting increasingly significant trends toward coordinated development.

Institutional mechanisms for regional cooperation have been established. In September 2014, Hong Kong, Guangdong, and Macao signed the Cooperation Agreement on Regional Air Pollution Control and Prevention to foster regional cooperation and enhance the regional air quality monitoring network. From 2014 to 2017, the three jurisdictions conducted the Guangdong-Hong Kong-Macao Joint Regional PM2.5 Study to bring continuous improvement to regional air quality. Yang et al. (2024) [60] proposed integrating policy quantification analysis into ecological security pattern construction for the GBA, demonstrating that policy adaptability has substantial impact on environmental security.

#### 2.5.3. Identified research gaps.

Despite this growing body of literature, several critical gaps remain: Lack of Quantitative Evaluation Frameworks. Research on Macao’s sustainability has primarily employed qualitative assessments or focused on outcome indicators rather than policy design quality. No studies have systematically applied structured policy evaluation frameworks such as the PMC index to assess Macao’s environmental policies. Limited Dynamic Assessment Methods. Existing research tends to provide static snapshots rather than tracking policy evolution over time. While studies document that the GBA has undergone rapid urbanization and environmental changes over four decades, systematic longitudinal analyses of how policies have adapted to these changes remain scarce. Insufficient Integration of Methods. Zhang et al. (2024) [61] identified the absence of common ecological targets and effective cross-border regulative measures as key problems in GBA environmental cooperation. yet no research has combined text mining approaches with quantitative policy consistency evaluation to address this gap systematically. Therefore, this study addresses these gaps by (1)Employing an integrated text mining and PMC index methodology to systematically evaluate Macao’s environmental policy design. (2)Providing dynamic assessment tracking policy evolution from 2010–2024. (3)Offering quantitative metrics for policy consistency and comprehensiveness adapted to Macao’s unique governance context. (4)Generating empirically-grounded recommendations for enhancing both policy design and regional cooperation mechanisms.

## 3 Text mining and thematic analysis

### 3.1 Data corpus and selection criteria

This study analyzes 12 environmental policy texts issued by the Macao Special Administrative Region government between 2010 and 2024. These policy texts were obtained from the official database of the Environmental Protection Bureau (DSPA) of the Macao Special Administrative Region to ensure data authenticity and comprehensiveness. This dataset covers the evolution of environmental policies since Macao’s return to China, laying a solid foundation for this research. The selection criteria were as follows: (1) policies issued between 2010 and 2024 (2) directly related to environmental management, pollution prevention and control, or energy (3) representing different types of policies such as regulations, plans, and standards to ensure diversity. The original texts were written in Chinese and Portuguese, but official English translations were used to ensure consistency in Natural Language Processing (NLP) analysis.

### 3.2. Text preprocessing

To conduct meaningful analysis, raw textual data needs to be preprocessed. This process includes text cleaning and normalization. Text classification and preprocessing are critical for ensuring analysis accuracy. Following best practices outlined by Danesh and Dastani (2023) [62], we implemented a multi-stage preprocessing pipeline including tokenization, stopword removal, and domain-specific filtering. Their work on COVID-19 publication classification demonstrates that careful preprocessing significantly improves downstream analysis quality, a principle we applied to our policy document analysis.

The specific steps are as follows:

(1) Tokenization: Breaking down text into individual words.
(2) Removing punctuation: Eliminating punctuation marks and special characters not needed for analysis.
(3) Removing stopwords: One of the most important steps is removing stopwords (e.g., “the”, “is”, “in” and other common words that are grammatically necessary but have relatively low semantic value). Using only standard stopword lists may not be sufficient in the context of specific domains. Therefore, this study first used the standard English stopword list provided by the Python Natural Language Toolkit (NLTK) library and expanded upon it.

I created a custom stopword list. This custom list includes words identified in preliminary analysis as meaningless in the context of this research (e.g., “conclude”, “specifically”, “government”), as well as routine administrative terminology that does not reflect substantive policy content (e.g., “regulation”, “article”). This customized approach enhances the accuracy of the analysis and ensures that the resulting keywords more precisely reflect the core content of Macao’s environmental policies.

### 3.3. High-frequency word analysis

**Fig 1:**
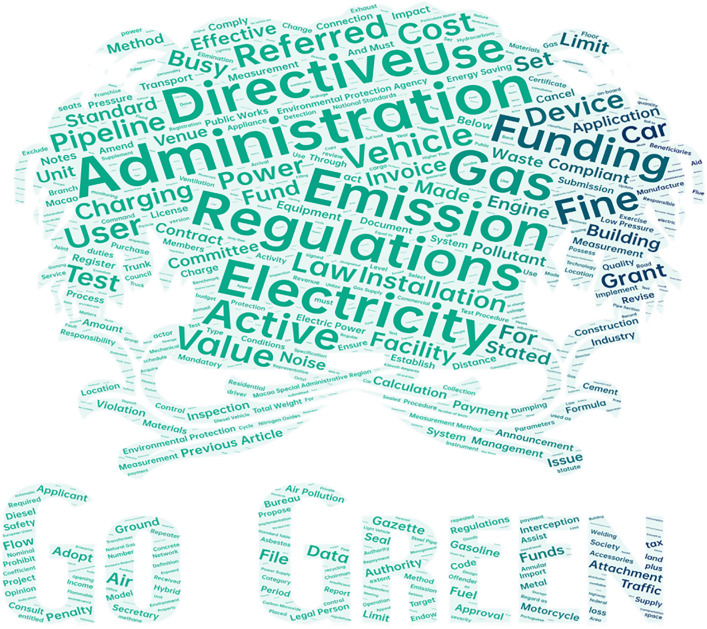
High Frequency Word Chart.

After preprocessing, text mining methods were used to identify important terms in policy documents. Further analysis of word frequency using the Term High-frequency word method highlights the importance. These terms, Policy text high frequency words statistics in Table 1, with a detailed visualization provided in Fig 1. These findings confirm that these terms not only occur frequently but also carry substantial importance as keywords in shaping the discourse of Macao’s environmental policies.

**Table 1:** Policy Text High-Frequency Words Statistics (Frequency > 100).

High frequency words	Frequency	High frequency words	Frequency
Administration	535	Value	130
Regulations	340	Installation	129
Gas	289	Facility	129
Electricity	285	Previous Article	127
Emission	278	Committee	124
Directive	258	Compliant	121
Funding	258	Building	119
Referred	250	Effective	116
Active	246	Law	116
Vehicle	215	Invoice	116
Charging	205	Busy	110
Pipeline	186	Grant	109
Use	182	Pollutant	106
Fine	178	Macao Special Administrative Region	106
Device	178	Stated	106
Power	162	Fund	105
Standard	157	Engine	104
Environmental Protection Agency	152	Management	103
User	151	Test	102
Cost	136	Car	102
Application	136	Contract	101

From the distribution characteristics of high-frequency words, Macao’s environmental policies present distinct management-oriented features. “Administration”, as the most frequent word, fully reflects the high emphasis on administrative management functions in policy texts. This phenomenon is consistent with findings in research literature that word frequency analysis can reflect the core concerns and policy orientation of policymakers.Combined with the high frequency of legal terms such as “Regulations” and “Directive”), it constructs a policy system framework with administrative management at its core and legal regulations as support.

In the field of environmental pollution control, high-frequency vocabulary shows obvious focusing characteristics. The high frequency of “Gas”, “Electricity”, and “Emission” reflects Macao’s environmental policies’ key focus on energy use and pollutant emission control. This vocabulary distribution pattern aligns with the method mentioned in the literature of identifying policy priority areas through high-frequency word analysis. Particularly noteworthy is the prominent position of transportation-related vocabulary such as “Vehicle” and “Charging”, revealing the unique challenges Macao faces as a high-density city in traffic pollution control.

From the perspective of policy tool application, economic means occupy an important position. “Funding”, as the second most frequent word after administrative management, combined with economic vocabulary such as “Cost” and “Grant”, reflects Macao’s environmental policies’ emphasis on promoting environmental protection goals through economic incentive mechanisms. In the regulatory enforcement dimension, the high frequency of words like “Fine”and “Inspection” demonstrates the strict enforcement characteristics of Macao’s environmental policies. These words form an organic combination with administrative management vocabulary, constructing a complete policy chain of “formulation-implementation-supervision”. As stated in the literature, semantic network analysis can reveal the structural relationships between high-frequency words, and the co-occurrence relationship between these regulatory vocabulary and management vocabulary reflects the systematization and rigor of policy implementation.Regarding infrastructure construction, vocabulary such as “Pipeline”, “Facility”, and “Installation” reflects Macao’s environmental policies’ emphasis on environmental infrastructure construction. This vocabulary distribution characteristic is consistent with the methodology mentioned in research that “word frequency statistics can identify important themes in policy texts”, reflecting policymakers’ attention to hardware facility investment.Particularly noteworthy is the appearance of regional and institutional vocabulary such as “Environmental Protection Agency”and “Macao Special Administrative Region”, which reflects the local characteristics and institutional arrangements of Macao’s environmental policies. This vocabulary feature echoes the viewpoint in the literature that “policy text analysis needs to consider regional characteristics and institutional background”.Comprehensive analysis shows that the high-frequency vocabulary of Macao’s environmental policies presents obvious hierarchical structure and internal connections. From management systems to specific measures, from economic means to technical support, it forms a multi-dimensional, all-round policy system. This vocabulary distribution pattern not only reflects Macao’s practical needs for environmental governance in limited space but also embodies its unique path of exploring environmental governance modernization under the “One Country, Two Systems” framework. Through in-depth analysis of these high-frequency words, it provides important textual evidence for understanding and evaluating the characteristics and effects of Macao’s environmental policies.

### 3.2 Semantic network topic modeling

To identify broader themes beyond individual words in policy documents, we applied semantic network analysis and topic modeling (as shown in Table 2), Social Network Semantic Graph. The table displays node-level properties (degree, weighted degree, modularity class) rather than pairwise co-occurrence frequencies.

**Table 2:** Social Network Semantic Graph.

Label	Degree	Weighted	Category	Label	Degree	Weighted	Category
Carbon Monoxide	24	78	1	Manufacture	101	850	3
System	103	608	4	Area	30	94	3
Exhaust	56	444	1	Venue	120	1082	3
Emission	192	3618	1	Fund	118	1692	5
Standard	198	1996	2	Statement	61	326	4
Gas	53	160	5	Treatment Plant	43	164	3
Nitrogen Oxides	30	92	1	Penalty	41	630	5
Pollutants	129	2030	1	Committee	126	1952	4
Regulations	325	4506	4	Installation	187	1618	3
Test	128	1300	2	Implementation	75	378	5
Methane	7	46	1	Laboratory	44	190	3
Hydrocarbons	26	140	1	Seal	63	314	2
Administration	344	5838	4	Process	25	104	3
Comply	147	840	4	Suggestion	61	370	4
Limit	80	1142	1	Mandatory	78	838	5
Particulate Matter	41	180	1	Levy	27	516	5
Affairs Bureau	101	786	5	Opinion	66	440	4
Measurement	86	512	2	Deduction	51	202	5
Fuel	106	508	1	Execution	126	1094	3
Dedicated	75	214	2	Directive	176	2350	4
Manager	42	378	5	Contractor	32	128	3
Representative	51	712	5	Instruction	28	262	5
Enterprise	46	234	4	Exclude	24	308	5
Meeting	29	98	5	Charges	107	1586	5
Protection	78	374	2	Value	120	1604	1
Deposit	31	128	4	Service	67	218	2
Information	54	660	1	Review	25	328	4
Storage	64	194	3	Approval	150	1304	1
Public Sector	43	148	3	Maintenance	33	118	2
Pressure Reducer	54	346	2	Sewage	51	182	3

Technical explanation: From this network, we calculated: (1) Degree: number of unique terms each word co-occurs with; (2) Weighted: sum of co-occurrence frequencies; (3)Category: thematic community assignment from Louvain clustering. These metrics reveal each term’s semantic centrality and thematic role.

**Fig 2:**
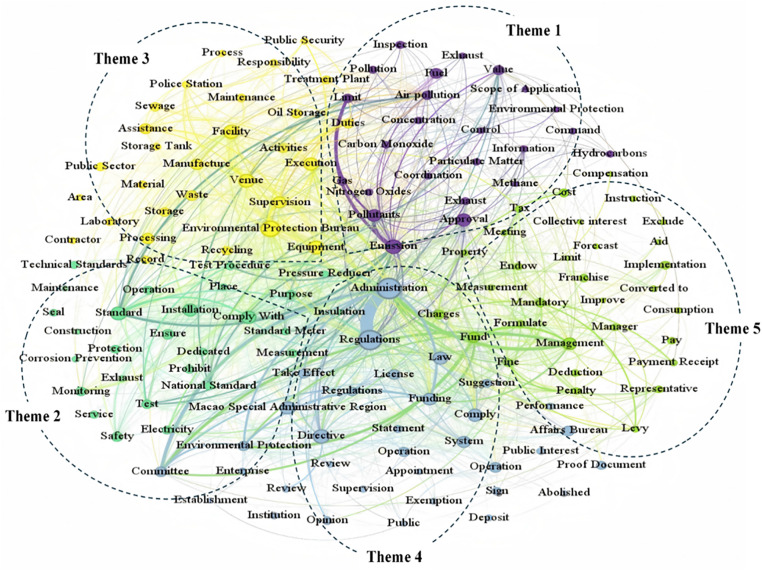
Thematic clustering of the semantic network.

A thematic clustering diagram (Fig 2), constructed using the semantic web co-occurrence matrix and a web semantic model, identifies five critical themes in the quantitative assessment of environmental policies in the Macao Special Administrative Region (Macao SAR). These themes collectively form the framework for Macao’s environmental protection initiatives and reflect the multidimensional efforts of the Macao SAR government in environmental governance.

#### Theme 1 environmental quality and pollutant emission control.

This theme emphasizes “pollutants”, “air”, “vehicles”, and “emissions”, highlighting the Macao government’s focus on controlling pollutant emissions, particularly from vehicles. The aim is to reduce traffic-related pollution and improve air quality across the region. This theme emphasizes pollutant emission control, particularly vehicular emissions. The evolution mechanism shows a shift from reactive measures to proactive strategies, indicating policy learning and maturation over time.

#### Theme 2 environmental monitoring and technical regulation.

This theme underscores terms like “power”, “monitoring”, and “electricity”, reflecting Macao’s prioritization of technical and scientific approaches to environmental monitoring. These efforts ensure the effective implementation of environmental policies and provide reliable data to support ongoing environmental protection measures. The prominence of monitoring-related terminology reveals Macao’s governance philosophy rooted in evidence-based policymaking and regulatory precision. The evolution mechanism demonstrates a progression from basic compliance monitoring to predictive and adaptive monitoring, indicating increasing technical sophistication and integration with regional monitoring networks in the Greater Bay Area.

#### Theme 3 environmental protection facilities.

Terms such as “installations”, “pipelines”, and “buildings” highlight Macao’s emphasis on strengthening environmental infrastructure. This focus includes enhancing pollution control capabilities and modernizing environmental facilities to meet contemporary needs. The infrastructure-centric vocabulary reflects Macao’s pragmatic approach to overcoming spatial constraints through technological intensification. The evolution mechanism shows a shift from isolated facility construction to networked infrastructure systems, indicating strategic reorientation from self-sufficiency to regional integration as the fundamental approach to infrastructure development.

#### Theme 4 legal framework and participation of associations.

This theme highlights the Macao government’s efforts to develop and enforce robust environmental laws while fostering collaboration with associations. The co-occurrence of terms related to legal frameworks and partnerships underscores the importance of institutional and cooperative approaches in advancing environmental protection. The evolution mechanism demonstrates progression from top-down regulatory frameworks to collaborative governance models, indicating maturation from command-and-control to participatory environmental governance that reflects growing civil society capacity and government confidence in shared decision-making.

#### Theme 5 application and management of environmental programs.

Key terms such as “application”, “funding”, “management”, and “fund” reflect Macao’s strategy to encourage community participation in environmental initiatives. By providing financial and resource support, the government aims to promote sustainable development and environmental stewardship within the community. The evolution mechanism shows a shift from hardware-focused subsidies to comprehensive behavioral change programs indicating strategic evolution from technology-push to demand-pull environmental policy that seeks to embed sustainable practices into business operations and household routines rather than simply financing one-time infrastructure improvements.

### 3.6. Integration of text mining and PMC index

The methodological innovation of this study lies in integrating two methods: text mining and the PMC index. This integration is based on a sequential explanatory mixed research design.(1) Exploratory Phase (Text Mining): As an initial step, text mining was used for exploratory analysis of the entire corpus of Macao’s environmental policy documents. As previously mentioned, during this phase, major concepts, priorities, and means in policy discourse were identified (high-frequency words, semantic themes). These qualitative findings empirically revealed “what” Macao’s policies focus on.(2) Evaluation Phase (PMC Index): Subsequently, we directly applied insights obtained from text mining to construct and validate the PMC index model. The PMC model requires a series of structured variables to evaluate policies. The results of text mining provided an empirical foundation for ensuring these variables are relevant and comprehensive in the Macao context. For example, text mining highlighted themes such as “pollutant emission management” and “financial management,” confirming the rationale for specifically including X_6_ (Policy Focus) and X_8_ (Policy Themes) in the variables of the PMC model.

Through this integration, this study was able to move beyond merely describing policy content or conducting context-free abstract assessments. Text mining established the assessment framework on a data foundation, while the PMC index quantitatively evaluated the systematization and consistency of identified themes in policy design. Thus, these two methods complemented each other, generating synergy between qualitative insights and quantitative rigor in the dynamic assessment of Macao’s environmental policies.

## 4. Quantitative policy assessment based on the PMC index model

### 4.1. Policy examples for PMC analysis

After text mining analysis of 36 policies, we selected 12 policies representing multiple aspects of Macao’s environmental governance for more detailed quantitative assessment. The selection of these policies was based on the following criteria: (1) covering diverse environmental domains including air, water, waste, energy, etc.; (2) representing policy developments from 2011 to 2022, capturing policy evolution; (3) policies considered to have significant impact on environmental outcomes. These policies originate from the legal department of the Macao Special Administrative Region and are summarized in Table 3.

**Table 3:** Summary of the sample of Macao’s environmental policy effectiveness evaluation.

Serial Number	Name	Issue No.	Year
1	Public Service Charging System for Electricity Supply (P1)	Administrative Regulation No. 25/2024	2024
2	Technical Specifications for Gas Facilities in Buildings (P2)	Administrative Regulation 27/2021	2021
3	Emission Standards of Air Pollutants for Concrete Manufacturing Industrial Sites and Regulations on Facilities Management (P3)	Administrative Regulation No. 22/2020	2021
4	Air Pollutant Emission Standards for Boilers in Industrial and Commercial Premises (P4)	Administrative Regulation No. 28/2021	2021
5	Construction Waste Management System (P5)	Administrative Regulation 22/2020	2020
6	Air Pollutant Emission Standards for Power Plants (P6)	Administrative Regulation 24/2019	2019
7	Air Pollutant Emission Standards for Sewage Treatment Plants (P7)	Administrative Regulation 37/2018	2018
8	Equipment and Vehicle Grant Scheme for the Recycling Industry (P8)	Administrative Regulation 32/2018	2018
9	Emission Limits and Measurement Methods for Pollutants Emitted from the Tailpipe of In-use Vehicles (P9)	Administrative Regulation 30/2016	2016
10	Prevention and Control of Environmental Noise (P10)	Administrative Regulation8/2014	2014
11	Requirements on Tailpipe Emission Standards to be Observed for Imported New Vehicles (P11)	Administrative Regulation No. 1/2012	2012
12	Funding Scheme for Environment-friendly and Energy-saving Products and Equipment (P12)	Administrative Regulation No. 22/2011	2011

This study evaluates the effectiveness of environmental policies in the Macao SAR by employing the Policy Modeling Consistency Index (PMC Index) and incorporating the Omnia Mobilis hypothesis proposed by Ruiz Estrada et al. [52]. he PMC index model, which utilizes binary values (zeros and ones) to quantify each variable, provides an objective framework for analyzing the strengths, weaknesses, and internal consistency of policies [53].

The selection of the 12 representative environmental policies was guided by several criteria to ensure comprehensive representation. These criteria included: (1) Diversity of environmental focus areas to capture the spectrum of Macao’s policy efforts, including pollution control, energy management, and public engagement; (2) Representation of different stages of policy evolution from 2010 to 2024 to include both foundational and contemporary policy measures; (3) Perceived impact and significance of the policies in shaping environmental outcomes; and (4) Availability of sufficient documentation to enable consistent application of the PMC index model and text mining analysis. The 12 selected policies are intended to serve as a representative cross-section of Macao’s environmental strategies, addressing key areas such as emissions control, energy efficiency, and waste management. However, it is acknowledged that certain specific areas—such as biodiversity conservation and community-specific initiatives—may have limited representation in this selection. While these 12 policies provide a broad overview of Macao’s environmental governance efforts, the complexity and multifaceted nature of environmental policy-making necessitate further research to include other areas that may also play a significant role in the overall sustainability strategy of Macao.

### 4.2. PMC index modeling and variable definition

Based on detailed analysis of relevant literature and the results of text mining, this study establishes a refined policy assessment framework consisting of 9 primary indicators and 50 secondary indicators. These indicators provide a comprehensive foundation for evaluating the effectiveness and scope of environmental policies in the Macao Special Administrative Region.

**Policy Type (X**_**1**_): Assesses the role and function of policies through 6 secondary aspects such as prediction, regulation, consultation, description, guidance, etc.
**Policy Timeliness (X**_**2**_): Focuses on the time frame of policies, including three categories: long-term (over 10 years), medium-term (5-10 years), and short-term (1-5 years).
**Policy Constraints (X**_**3**_): Includes 11 secondary indicators such as regulations, emission limits, supervision, penalties, standards implementation, funding, subsidies, etc., used to evaluate policy incentive and constraint mechanisms.
**Policy Domain (X**_**4**_): Assesses the application areas and scope of impact of policies through 5 aspects such as quality management, green development, risk response, citizen participation, etc.
**Policy Evaluation (X**_**5**_): Evaluates the rationality and scientific basis of policies through 4 aspects such as sufficient evidence, clear objectives, scientific planning, etc.
**Policy Focus (X**_**6**_): Covers 10 important aspects such as environmental protection, energy management, urban greening, waste treatment, etc., evaluating priority areas.
**Policy Scope (X**_**7**_): Assesses the geographical and administrative applicability of policies, emphasizing their comprehensive application range and universal relevance.
**Policy Themes (X**_**8**_): Explores the emphasis on major themes such as pollutant emission management, energy efficiency, environmental regulations, etc.
**Policy Openness (X**_**9**_): Evaluates policy transparency, ensuring public access to information, thereby fostering trust and inclusiveness.

To clearly distinguish between X6 (Policy Focus) and X8 (Policy Themes), this study defines them as follows. “Policy Focus” refers to the broad strategic priority areas pursued by policies (e.g., environmental protection as an overall goal). “Policy Themes,” on the other hand, are more specific and operational issues explicitly articulated in policy texts (e.g., pollutant emission monitoring, financial management). Through this distinction, we can assess both the strategic intent and specific content of policies.To ensure the legitimacy of the selection and definition of each variable, we constructed a detailed indicator system. Table 4 shows the primary and secondary variables, their assessment criteria, and the literature or theoretical basis supporting their adoption.

**Table 4:** Scoring criteria for secondary variables.

Primary Variables	Code	Secondary Variables (Code)	Evaluation Criterion	Justification
Policy Type	**X**_**1**_	Forecast (X_**1:1**_)	Whether the policy is predictive	Standard policy function
		Regulation (X_**1:2**_)	Whether the policy is regulatory	
		Recommendation (X_**1:3**_)	Whether the policy is recommendatory	
		Description (X_**1:4**_)	Whether the policy is descriptive	
		Guidance (X_**1:5**_)	Whether the policy is guiding	
		Other (X_**1:6**_)	Does the policy involve content other than the above	
Policy Timeliness	**X**_**2**_	Long-term (X_**2:1**_)	Does the policy involve long-term content (10 + years)	Time horizon is a key policy design element
		Medium-term (X_**2:2**_)	Does the policy involve medium-term content (5–10 years)	
		Short-term (X_**2:3)**_	Whether the policy involves short-term content (1–5 years)	
Policy Constraints	**X**_**3**_	Regulatory Standards (X_**3:1**_)	Whether the policy involves compliance with legal requirements for environmental protection	Derived from text mining results
		Emission Limits (X_**3:2**_)	Whether the policy involves numerical limits for pollutant emissions	
		Environmental Monitoring (X_**3:3**_)	Whether the policy involves regular environmental monitoring	
		Penalties for Environmental Violations (X_**3:4**_)	Whether the policy involves penalties for environmental offences	
		Enforcement of Environmental Standards (X_**3:5**_)	Whether the policy involves the implementation of environmental protection standards	
		Funding and Subsidies (X_**3:6**_)	Whether the policy involves funding and subsidizing energy conservation projects	
		Management of Environmental Protection Funds (X_**3:7**_)	Whether the policy involves environmental protection funds	
		Environmental Funding and Subsidies (X_**3:8**_)	Whether the policy involves funding and subsidies for environmental protection	
		Public Participation (X_**3:9**_)	Whether the policy involves public environmental incentives	
		Environmental Protection Environment (X_**3:10**_)	Whether the policy involves environmental protection to enhance public awareness of environmental protection	
		Others (X_**3:11**_)	Whether the policy involves other essential elements	
Policy area	**X**_**4**_	Quality Control (X_**4:1**_)	Whether the policy involves environmental quality and pollution control policies	Based on policy typology literature
		Green Development (X_**4:2**_)	Whether the policy involves green development and sustainable urban planning	
		Risk Response (X_**4:3**_)	Is the policy related to environmental risk and adaptation policy	
		Public Participation (X_**4:4**_)	Whether the policy is on environmental environment and public participation	
		Others (X_**4:5**_)	Whether the policy involves other contents than the above	
Policy Evaluation	**X**_**5**_	Sufficient Basis (X_**5:1**_)	Whether the policy is based on sufficient evidence	Based on policy typology literature
		Clear Objectives (X_**5:2**_)	Whether the policy objectives are clear	
		Scientific Programme (X_**5:3**_)	Whether the policy programme is scientific	
		Others (X_**5:4**_)	Whether the policy involves other contents than the above	
Policy Focus	**X**_**6**_	Environmental protection and pollution control (X_**6:1**_)	Whether the policy attaches importance to pollutant emission standards and environmental monitoring	Core theme from text mining
		Energy Management and Sustainable Development (X_**6:2**_)	Whether the policy attaches importance to the structure of energy consumption and the promotion of renewable energy sources	
		Urban Greening and Ecological Construction (X_**6:3**_)	Whether the policy attaches importance to urban green space planning, construction and management	
		Waste Treatment and Resource Recovery (X_**6:4**_)	Whether the policy attaches importance to waste classification, treatment and recycling	
		Water Resources Protection and Water Pollution Control (X_**6:5**_)	Whether the policy attaches importance to pollution prevention and control of water resources	
		Climate Change and Environmental Adaptation (X_**6:6**_)	Whether the policy attaches importance to the impacts of climate change and countermeasures	
		Environmental Environment and Public Participation (X_**6:7**_)	Whether the policy attaches importance to the environment in raising public awareness of environmental protection	
		Environmental Regulation and Policy Development (X_**6:8**_)	Whether the policy attaches importance to assessing the effectiveness of implementing environmental laws and regulations.	
		Environmental Health and Risk Management (X_**6:9**_)	Does the policy emphasize the impact of environmental pollution on public health?	
		Others (X_**6:10**_)	Whether the policy has other priorities	
Policy Levels	**X**_**7**_	Provinces (X_**7:1**_)	Does the policy include provinces?	Based on policy literature
		Autonomous regions and municipalities (X_**7:2**_)	Does the policy include autonomous regions and municipalities	
		Ministries and Departments (X_**7:3**_)	Does the policy include ministries	
		Special Administrative Regions (X_**7:4**_)	Whether the policy covers particular administrative regions	
		Others (X_**7:5**_)	Whether the policy involves other than the above	
Policy Topics	**X**_**8**_	Pollutant Discharge Management (X_**8:1**_)	Whether the policy subject includes monitoring pollutant emissions	Specific topic identified in text mining
		Energy Efficiency Utilisation (X_**8:2**_)	Whether the policy theme focuses on improving energy efficiency	
		Environmental Protection and Regulations (X_**8:3**_)	Whether the policy subject includes environmental protection laws and regulations	
		Architecture and Urban Planning (X_**8:4**_)	Whether the policy subject includes environmental impact and urban planning	
		Environmental Protection and Energy Conservation and Fund Management (X_**8:5**_)	Whether the policy topic covers the management of environmental funds	
		Others (X_**8:6**_)	Whether the policy theme includes others	
Policy Disclosure	**X**_**9**_	Policy Disclosure (X_**9:1**_)	Is the policy open to the public	Transparency is a key governance principle

#### 4.2.1. Expert consultation and ethical considerations.

To ensure that the PMC index variables adapt to Macao’s unique policy environment, we consulted with domain experts. In May 2023, we conducted semi-structured interviews with 5 experts, including 3 senior environmental policy scholars from Macao Polytechnic University, and 2 environmental consulting advisors with over 10 years of regional experience. The purpose of this consultation was to validate the effectiveness of the variable set and to capture the nuances specific to Macao’s environmental context through refined indicators.

Regarding ethical considerations, this study followed the guidelines of the university’s Institutional Review Board (IRB). The interviews focused on experts’ opinions about the policy framework rather than personal information about the experts, and the data collected was intended to inform the construction of research tools rather than directly analyze expert views. Therefore, our university IRB determined that this project did not constitute human research requiring full IRB review and approved the exemption application.

### 4.3. Model construction and calculation

Using the MSAR environmental policy evaluation indicator system, a multi-input, multi-output table can be easily derived for analyzing the content of various policy texts. This table serves as a structured data analysis framework for quantitatively assessing variables in policy texts. To maintain balance between variables, the assessment primarily uses binary values. Based on this table, the PMC index value for each policy can be calculated.

The environmental policy texts of the Macao Special Administrative Region were analyzed, and values for secondary variables were assigned according to formulas (1) and (2). Here, the values of secondary variables range from 0 to 1. The values of primary variables were calculated using formula (3), with scores ranging from 1 to 9. Finally, formula (4) calculates the PMC index of Macao’s environmental policies by summing the primary variable scores for each policy.

This approach provides a systematic and quantitative means of evaluating the effectiveness of environmental policies, ensuring a balanced and objective analysis while offering insights into areas for potential improvement.


X~N[0,1; (1)

X={XR:[0~1]} (2)

$$Xt\left\{(\sum_{j=1}^{n}\frac{X_{tj}}{T(X_{tj})}\;\right\}\;\left(t=1,2,3,4,5,6,7,8,9\right)\;$$ (3)

$$PMC=\left[\begin{array}{c} \;X_{1}\;(\;\sum_{i=1}^{6}\frac{X_{1a}}{\text{6}})+X_{2}\;(\sum_{i=1}^{3}\frac{X_{2b}}{\text{3}})+\;X_{3}\;(\sum_{i=1}^{10}\frac{X_{3c}}{10})+\\ X_{4}\;(\;\sum_{i=1}^{5}\frac{X_{4d}}{\text{5}})+X_{5}\;(\sum_{i=1}^{4}\frac{X_{5e}}{\text{4}})+\;X_{6}\;(\sum_{i=1}^{10}\frac{X_{6f}}{10})+\\ \;X_{7}\;(\;\sum_{i=1}^{5}\frac{X_{7j}}{\text{5}})+X_{8}\;(\sum_{i=1}^{6}\frac{X_{8h}}{\text{6}})+\;X_{9}\;(\sum_{i=1}^{1}\frac{X_{9i}}{\text{1}}) \end{array}\right]\;\;$$ (4)

**Fig 3:**
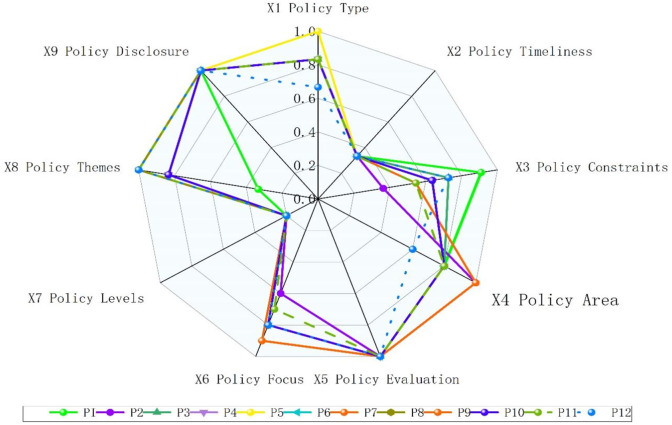
Evaluation of the Effectiveness of Environmental Policies in the Macao SAR.

Using the PMC index calculation method in conjunction with text mining, the input-output table for the 12 Macao environmental policy portfolios is derived, as shown in Table 5. Policies are evaluated and rated according to scores and evaluation criteria detailed in Table 6. Subsequently, the PMC index for each environmental policy is calculated and summarized in Fig 3, referred to as the Macao Environmental Policy Score Table. In line with the specified criteria and requirements, the scores for each policy dimension are visually represented in Fig 3, providing a clear depiction of the performance across various dimensions of Macao’s environmental policies.

**Table 5:** Policy rating scale.

Score	10-8	7−5	4−2	2−0
Ratings	Excellent	Good	Acceptable	Unacceptable

**Table 6:** Scores of the 12 environmental policies of Macao.

Policy Dimension	P1	P2	P3	P4	P5	P6	P7	P8	P9	P10	P11	P12
X_1_ Policy Type	0.8333	0.8333	0.8333	0.8333	1.0000	0.8333	0.8333	0.8333	0.8333	0.8333	0.8333	0.6667
X_2_ Policy Timeliness	0.3333	0.3333	0.3333	0.3333	0.3333	0.3333	0.3333	0.3333	0.3333	0.3333	0.3333	0.3333
X_3_ Policy Constraints	0.9091	0.3636	0.7273	0.6364	0.6364	0.6364	0.5455	0.6364	0.6364	0.6364	0.5455	0.7273
X_4_ Policy Area	0.8000	1.0000	0.8000	0.8000	0.8000	0.8000	1.0000	0.8000	0.8000	0.8000	0.8000	0.6000
X_5_ Policy Evaluation	1.0000	1.0000	1.0000	1.0000	1.0000	1.0000	1.0000	1.0000	1.0000	1.0000	1.0000	1.0000
X_6_ Policy Focus	0.6000	0.6000	0.8000	0.8000	0.8000	0.8000	0.9000	0.8000	0.9000	0.8000	0.7000	0.8000
X_7_ Policy Levels	0.2000	0.2000	0.2000	0.2000	0.2000	0.2000	0.2000	0.2000	0.2000	0.2000	0.2000	0.2000
X_8_ Policy Themes	0.3333	0.8333	0.8333	0.8333	0.8333	0.8333	1.0000	1.0000	0.8333	0.8333	1.0000	1.0000
X_9_ Policy Disclosure	1.0000	1.0000	1.0000	1.0000	1.0000	1.0000	1.0000	1.0000	1.0000	1.0000	1.0000	1.0000
PMC Index	**6.0091**	**6.1636**	**6.5273**	**6.4364**	**6.6030**	**6.4364**	**6.8121**	**6.8030**	**6.5364**	**6.4364**	**6.4121**	**6.6273**
Policy Effectiveness	**Good**	**Good**	**Good**	**Good**	**Good**	**Good**	**Good**	**Good**	**Good**	**Good**	**Good**	**Good**

To ensure the robustness of the PMC index-based policy evaluations, we have incorporated a sensitivity analysis to examine the influence of changing the weightings of different variables on the overall policy scores. This analysis aims to assess the extent to which variations in variable weights impact the consistency and effectiveness ratings of Macao’s environmental policies. By doing so, we can evaluate whether certain variables disproportionately influence the outcomes and identify areas that require careful consideration during policy assessment. We have now strengthened the integration between our text mining findings and PMC index results. In Table 7 Mapping The Correspondence.

**Table 7:** Mapping The Correspondence.

Text Mining Theme	Related PMC Variables	Score Range	Interpretation
Theme 1: Pollutant Control	X6:1, X8:1, X3:2	0.60-0.90	Strong focus, variable implementation
Theme 2: Technical Monitoring	X6:1, X4:1, X3:3	0.80-1.00	Consistently high priority
Theme 3: Infrastructure	X6:3, X3:1	0.60-0.80	Moderate emphasis
Theme 4: Legal Framework	X1, X3:1, X3:4	0.55-1.00	Well-established
Theme 5: Program Management	X3:6- X3:8, X9	0.64-1.00	High transparency, Variable funding

To conduct the sensitivity analysis, we adjusted the weights of the nine primary variables by ±10% and recalculated the PMC indices for all twelve policies included in the study. The goal was to determine whether significant changes in the weights of these variables would result in substantial shifts in the final policy scores and rankings. The sensitivity analysis revealed that the rankings of policies remained generally consistent, even when primary variable weights were varied by ±10%. The sensitivity analysis has provided important insights into the robustness of the PMC index model used for evaluating Macao’s environmental policies.

To enhance the visualization of the PMC index results, a PMC surface can be created once the PMC index has been calculated. Given that there are nine first-level variables, these are organized into a 3 × 3 matrix for the surface representation. The calculation of the PMC surface is performed using Equation (5), which provides a structured approach to mapping the results into a visual format.


$$PMC=\left(\begin{array}{ccc} {}*20lX_{1} & X_{2} & X_{3}\\ X_{4} & X_{5} & X_{6}\\ X_{7} & X_{8} & X_{9} \end{array}\right)\;$$ (5)

### 4.4 Quantitative results assessment

#### 4.4.1 Evaluation of the perfect effectiveness of Macao’s environmental policy.

The selected environmental policies represent relatively excellent and comprehensive policies issued by the Macao SAR. The data analysis reveals distinct characteristics and differences in the scores of 12 policies (P1 to P12) across 9 dimensions. All policies achieve perfect scores in policy evaluation (X_5_) and policy disclosure (X_9_), indicating generally well-established assessment mechanisms and information transparency; however, they consistently score low in policy levels (X_7_) and timeliness (X_2_), reflecting common shortcomings in these areas. The comprehensive PMC indices range from 6.0091 to 6.8121, with P7 and P8 performing best, P1 scoring lowest, and later policies generally outperforming earlier ones. Dimensional differences are primarily manifested in policy constraints (X_3_), policy themes (X_8_), and policy focus (X_6_), where P5 uniquely achieves a perfect score in policy type, P2 and P7 excel in policy area, and P7, P8, P11, and P12 reach the highest level in policy themes. These data not only reflect the strengths and weaknesses of each policy but also reveal a trend of gradual improvement in the policy-making process, providing clear direction for future policy refinement.

#### 4.4.2 Evaluation of excellent effectiveness of environmental policies in Macao.

Our comprehensive evaluation of Macao’s environmental policies P1, P2, P3, P4, P5, P6, P7, P9, P10, and P11 demonstrates their excellent effectiveness across various dimensions. These policies exhibit diverse scores in areas such as policy type, timeliness, constraints versus incentives, domain importance, focus of policy evaluation, policy hierarchies and themes, and policy openness. The variation in scores across these dimensions highlights the unique strengths and areas of focus for each policy, reflecting the multidimensional approach of Macao’s environmental governance.

Policy P1: The *Public Service Charging System for Electricity Supply in Macao* achieves a PMC value of 6.009, highlighting its strong incentive characteristics for improving electricity supply services. While its focus and thematic importance are average, the policy excels in openness, demonstrating a commitment to balance and transparency in enhancing service efficiency; Policy P2: The *Technical Code for Gas Facilities of Buildings in Macao* also attains a PMC value of 6.009. This policy places a stronger emphasis on importance, with full openness and transparency. It establishes stringent specifications for ensuring the safety and compliance of building gas facilities; Policy P3: The *Macao Regulations on the Emission Standards of Air Pollutants and Management of Facilities of Industrial Sites Involving Concrete Manufacturing* achieves a PMC value of 6.5272, reflecting its high importance in environmental protection. This policy demonstrates a well-balanced mix of constraints and incentives, along with a thorough evaluation. It scores particularly high in policy focus and transparency, emphasizing its comprehensive approach; Policy P4: The *Air Pollutant Emission Standards for Boilers in Industrial and Commercial Sites in Macao* exhibits slightly weaker performance in the constraints and incentives dimension. However, it remains an essential component of Macao’s environmental policy framework; Policy P5: The *Construction Waste Management System in Macao* emphasizes the theme dimension, reflecting the government’s growing concern for effective waste material management; Policy P6: The *Air Pollutant Emission Standards for Power Plants in Macao* stands out for its clear policy focus, addressing critical issues related to air quality management; Policy P7: The *Air Pollutant Emission Standards for Wastewater Treatment Plants in Macao* achieves top ratings in policy areas and themes, underscoring its importance in advancing environmental protection; Policy P9: The *Emission Limits and Measurement Methods for Pollutants Emitted from the Exhaust Gases of In-use Vehicles in Macao* places significant emphasis on policy focus, targeting air quality improvements through stricter emissions control; Policy P10: The *Prevention and Control of Environmental Noise in Macao* also highlights its policy focus, addressing noise pollution issues as a key concern in urban environments; Policy P11: The *Regulations on Tailpipe Emission Standards to be Complied with by New Motor Vehicles Imported into Macao* is particularly relevant for newly imported vehicles. This policy underscores a prominent policy focus, reinforcing Macao’s commitment to reducing vehicular emissions.

Macao’s most effective environmental policies primarily focus on environmental protection and energy management, showing high levels of policy evaluation and openness. However, there are notable differences between policies in dimensions such as constraints and incentives, policy focus, and policy areas. These variations likely reflect the specific objectives of each policy and the urgency of their implementation, highlighting the tailored approach of Macao’s environmental governance framework.

In summary, from 2010 to 2024, Macao’s environmental policies have evolved from addressing immediate concerns to implementing comprehensive, forward-looking strategies. This progression has been influenced by economic development, technological innovation, regional collaboration, public participation, and integrated policy management, collectively enhancing the effectiveness of environmental governance in Macao.

### 4.5. Sensitivity analysis

To ensure the robustness of policy assessments based on the PMC index, we incorporated sensitivity analysis to study the impact of different variable weights on overall policy scores. This analysis aims to evaluate the extent to which fluctuations in variable weights affect the consistency and effectiveness of evaluating Macao’s environmental policies.

To conduct the sensitivity analysis, we adjusted the weights of the 9 primary variables by ±10% and recalculated the PMC index for the 12 studied policies. The goal was to determine whether substantially changing the weights of these variables would have a material impact on the final policy scores and rankings.

The results of the sensitivity analysis indicate that even with ±10% fluctuations in the weights of the primary variables, policy rankings remain largely consistent.

## 5. Conclusion and policy recommendations

### 5.1 Conclusion

This study investigated the policy conditions of the Macao Special Administrative Region government after its return through text mining and PMC index analysis, providing quantitative assessments of 12 representative environmental policies. Text mining revealed that Macao’s environmental policies primarily focus on core themes such as regulation and governance, energy and emissions, incentives and sanctions, infrastructure and technology, and transportation and vehicles. The PMC index analysis results indicate that the evaluated policies were all rated at an “acceptable” level, showing reasonable policy design, but with room for improvement in aspects such as policy timeliness (lack of long-term planning) and scope (limitations in regional cooperation).

This study makes several important contributions.Methodological Contribution: This study developed and applied an innovative framework integrating text mining and the PMC index model for dynamic assessment of environmental policies in the unique governance environment of a Special Administrative Region. This mixed research approach combines data-driven discovery of policy content with systematic quantitative assessment of policy design, surpassing traditional evaluation methods.This study conducted the first comprehensive and quantitative assessment of Macao’s environmental policies since its return. This empirically clarified specific strengths (e.g., effectiveness of evaluation, openness) and weaknesses (e.g., lack of long-term vision) of policies that were previously understood mainly in descriptive ways.This study provides evidence-based tools and specific recommendations for Macao’s policymakers to refine existing policies and guide future environmental governance. For example, the research identifies areas requiring specific interventions for “policy constraints” and “policy focus” aspects that received lower PMC scores.

The assessment results of this study provide valuable insights for the continuous improvement of environmental policies in the Macao Special Administrative Region. Policymakers should address weaknesses identified in the PMC model, particularly strengthening long-term strategic planning and regional cooperation.International best practices provide valuable insights into areas where Macao’s environmental policies can be further developed.

Germany’s Energy Transition (Energiewende): Through sustained policy support, such as the Renewable Energy Act (EEG), Germany has successfully increased the proportion of renewable energy significantly. In 2023, renewable energy accounted for more than half of Germany’s electricity consumption for the first time, setting a historic record. Macao can learn from Germany’s experience about the importance of long-term incentive mechanisms and stable legal frameworks for accelerating the introduction of renewable energy.

Japan’s Circular Economy Framework: Japan was one of the first countries to introduce circular economy policies, beginning with the “Basic Act for Establishing a Sound Material-Cycle Society” in 2000. Japan’s framework emphasizes Extended Producer Responsibility (EPR) through specific laws such as the Container and Packaging Recycling Law and the Home Appliance Recycling Law, and thoroughly manages waste throughout product lifecycles. The implementation of the “Act on Promotion of Resource Circulation for Plastics” in 2022 further strengthened these efforts. Macao can reference this comprehensive and targeted legal approach to enhance waste management and resource efficiency.By learning from these international cases, Macao can move toward a more advanced environmental policy framework that addresses both regional needs and global sustainability challenges.

The quantitative methods adopted in this study provide a structured and replicable means of evaluating policy consistency and focus, but this approach has inherent limitations. Particularly in capturing the complete picture of policy effectiveness, it is difficult to encompass stakeholders’ subjective experiences and challenges encountered during implementation. The absence of qualitative insights may limit understanding of how policies are perceived and accepted by the public and relevant industries.To more comprehensively assess policy effectiveness, it is recommended to employ qualitative methods, such as stakeholder interviews, in addition to quantitative analysis. Interviews with policy implementers, industry representatives, local residents, and non-governmental organizations would provide valuable insights into subjective experiences, challenges, and successes related to policies. These perspectives help identify gaps that cannot be discovered through quantitative analysis alone and provide a more complete comprehensive picture of the strengths and weaknesses of Macao’s environmental policy framework. By adopting a mixed research approach, we can gain a more comprehensive understanding of the effectiveness of Macao’s environmental policies.

### 5.2 Policy recommendations

To address the significant deficiency in Policy Timeliness (X2). Propose a three-tiered temporal planning framework. First, Macao should establish rolling five-year environmental master plans with mandatory review cycles, institutionally linked to the SAR government’s five-year development plans to ensure policy continuity and political commitment. Second, the government should develop comprehensive 2030 and 2050 environmental target frameworks with specific, measurable milestones for carbon neutrality, air quality standards, and waste reduction targets that align with China’s national environmental goals while accommodating Macao’s unique characteristics as a Special Administrative Region. Third, long-term policy implementation requires stable financial support; therefore, we recommend creating dedicated long-term funding commitments through establishment of an Environmental Development Fund with multi-year budget allocations insulated from annual fiscal volatility.

Regarding the weakness in Policy Scope (X7), reflecting Macao’s limited regional integration, propose comprehensive measures to strengthen cross-border environmental cooperation within the Greater Bay Area framework. The first priority involves formalizing environmental policy coordination mechanisms through three specific institutional arrangements: establishing quarterly environmental policy coordination meetings with Guangdong and Hong Kong authorities to ensure regular communication and joint planning; creating joint air quality monitoring networks with shared real-time data platforms that transcend administrative boundaries; and harmonizing emission standards for cross-border vehicles and vessels to eliminate regulatory arbitrage and improve overall regional environmental performance. Beyond institutional mechanisms, Macao should pursue tangible cross-border environmental infrastructure projects, including joint waste treatment facilities with capacity-sharing agreements to address land constraints, integrated renewable energy grid connections to enhance energy security and sustainability, and coordinated coastal water quality management systems leveraging the shared marine environment of the Pearl River Delta.

Finally, to operationalize regional cooperation, Recommend implementing three categories of policy pilot programs: cross-recognition of environmental certifications across the Greater Bay Area to reduce bureaucratic barriers and promote best practices; joint green procurement standards for government projects to create economies of scale and market transformation; and coordinated environmental impact assessment procedures for regional infrastructure projects to ensure comprehensive evaluation of transboundary environmental effects. These specific, actionable recommendations transform the general observation that policies require “strengthening long-term planning and regional cooperation” into concrete measures with clear implementation pathways, institutional arrangements, and expected outcomes.

### 5.3 Applicability analysis of international best practices to Macao

Germany’s Energiewende offers important lessons regarding the effectiveness of long-term policy frameworks and renewable energy incentives. However, direct application to Macao faces substantial structural constraints that necessitate careful adaptation. The most fundamental challenge stems from Macao’s severely limited territory, which precludes the large-scale solar farms and wind installations that characterize Germany’s renewable energy infrastructure. To address this constraint, Macao must focus on spatially efficient alternatives, including building-integrated photovoltaics that utilize existing vertical surfaces, offshore wind collaboration with neighboring regions that leverage shared marine areas, and cross-border renewable energy procurement agreements within the Greater Bay Area framework that allow Macao to meet environmental targets through regional cooperation. Japan’s comprehensive Extended Producer Responsibility framework presents a particularly relevant model for Macao, though implementation requires substantial adaptation to account for institutional and scale differences. The structural similarities between Japan and Macao are striking. Both jurisdictions face severe land constraints, extraordinarily high population densities, and limited capacity for traditional landfill-based waste management.

This applicability analysis demonstrates that while international best practices provide valuable conceptual frameworks and proven policy instruments, successful policy transfer requires critical contextual analysis that accounts for Macao’s unique characteristics: land constraints, Special Administrative Region governance structure, tourism-dependent economic model, and strategic position within the Greater Bay Area regional system. Effective borrowing policy is therefore not imitation but rather adaptive translation that preserves core principles while fundamentally reconceptualizing implementation mechanisms to fit local realities.
